# Prevalence of plasmid-mediated AmpC beta-lactamases in Enterobacteria isolated from urban and rural folks in Uganda

**DOI:** 10.12688/aasopenres.13165.1

**Published:** 2020-11-30

**Authors:** Christine F Najjuka, David Patrick Kateete, Dennis K Lodiongo, Obede Mambo, Chunderika Mocktar, William Kayondo, Hannington Baluku, Henry M Kajumbula, Sabiha Y Essack, Moses L Joloba

**Affiliations:** 1 Department of Medical Microbiology, Makerere University College of Health Sciences, Kampala, Uganda; 2 Department of Immunology & Molecular Biology, Makerere University College of Health Sciences, Kampala, Uganda; 3 Ministry of Health Public Health Laboratory, National Blood Bank and Transfusion services Centre, Juba, Sudan; 4 Rumbek Health Science Institute, Lakes State, Sudan; 5 Antimicrobial Research Unit, School of Health Sciences, University of KwaZulu Natal, Westville, Durban, South Africa; 6 Makerere University Walter Reed Project, Box 16524, Kampala, Uganda

**Keywords:** Enterobacteriaceae, Escherichia coli, Klebsiella, Urban-Rural, Kampala-Uganda

## Abstract

**Background**: AmpC beta-lactamase-producing bacteria are associated with increased resistance to third-generation cephalosporins. Here, we describe plasmid-mediated AmpC beta-lactamase-producing enterobacteria isolated from urban and rural dwellers in Uganda.

**Methods**: Stool and urine from 1,448 individuals attending outpatient clinics in Kampala and two rural districts in central Uganda were processed for isolation of *Escherichia coli* and Klebsiella. Following antibiotic susceptibility testing, cefoxitin resistant isolates, and amoxicillin/clavulanate resistant but cefoxitin susceptible isolates, were tested for AmpC beta-lactamase production using the cefoxitin-cloxacillin double-disc synergy test. Carriage of plasmid-mediated AmpC beta-lactamase-encoding genes (pAmpC) and extended spectrum beta-lactamase (ESBL) encoding genes was determined by PCR.

**Results**: Nine hundred and thirty *E. coli *and 55 Klebsiella were recovered from the cultured samples, yielding 985 isolates investigated (one per participant). One hundred and twenty-nine isolates (13.1%, 129/985) were AmpC beta-lactamase producers, of which 111 were molecularly characterized for pAmpC and ESBL gene carriage. pAmpC genes were detected in 60% (67/111) of the AmpC beta-lactamase producers; pAmpC genes were also detected in 18 AmpC beta-lactamase non-producers and in 13 isolates with reduced susceptibility to third-generation cephalosporins, yielding a total of 98 isolates that carried pAmpC genes. Overall, the prevalence of pAmpC genes in cefoxitin resistant and/or amoxicillin/clavulanate resistant *E. coli *and Klebsiella was 59% (93/157) and 26.1% (5/23), respectively. The overall prevalence of pAmpC-positive enterobacteria was 10% (98/985); 16.4% (45/274) in Kampala, 6.2% (25/406) Kayunga, and 9.2% (28/305) Mpigi. Ciprofloxacin use was associated with carriage of pAmpC-positive bacteria while residing in a rural district was associated with protection from carriage of pAmpC-positive bacteria.

**Conclusion**: pAmpC beta-lactamase producing enterobacteria are prevalent in urban and rural dwellers in Uganda; therefore, cefoxitn should be considered during routine susceptibility testing in this setting.

## Introduction

Enterobacteriaceae is a family of Gram-negative bacteria that inhabit the mammalian gut and includes the leading causes of community- and hospital-acquired infections ^1^. Enterobacteriaceae have become increasingly resistant to antibiotics, especially beta (β)-lactam agents, the mainstay of treatment for infections caused by them. One of the main mechanisms underlying resistance to β-lactam antibiotics among the enterobacteriaceae are the AmpC β-lactamases. These enzymes are clinically relevant as they confer resistance to most β-lactam antibiotics, except the fourth-generation cephalosporins and carbapenems ^2– 4^.

AmpC β-lactamases are chromosomally encoded in most species of the enterobacteriaceae, particularly *Citrobacter freundii, Enterobacter*, *Morganella morganii*, *Hafnia alvei*, *Aeromonas* and *Serratia* spp. ^3^. However, *Escherichia coli* and other enterobacteria, notably *Proteus mirabilis*, *Salmonella* and *Klebsiella* spp., can acquire plasmid-encoded AmpC β-lactamases (pAmpC), which are highly transferable between species. Note that *Proteus mirabilis*, *Salmonella* and *Klebsiella* spp. lack chromosomally-encoded AmpC enzymes, while a chromosomally-encoded AmpC β-lactamase occurs in *E. coli* but it is expressed at low basal levels due to presence of a weak promoter and attenuator, which makes *E. coli* susceptible to cephamycins (e.g. cefoxitin, cefotetan) ^2, 5, 6^. Acquisition of pAmpC β-lactamases by species like *E. coli* and *Klebsiella pneumoniae* ( *K. pneumoniae*) is worrying as it enables an efficient spread of extended resistance in bacteria and ultimately their spread in the community ^3^. Furthermore, the widespread use of cephamycins and β-lactamase inhibitor combinations (e.g. clavulanic acid/amoxicillin and tazobactam/piperacillin) has contributed to selection of pAmpC β-lactamase producing strains worldwide ^7, 8^.

Once they have colonized the gut, pAmpC β-lactamase producing strains may initiate an infection at various anatomical sites ^9^. In low-income countries where such infections are empirically treated with third-generation cephalosporins, failure to detect AmpC β-lactamase-related resistance may lead to treatment failure. Moreover, the cut-off / break points for the disc diffusion / minimum inhibitory concentrations (MICs) may not detect AmpC β-lactamase production and resistance to third-generation cephalosporins. Carbapenems are currently the only effective drugs against infections caused by AmpC β-lactamase producing bacteria, since these bacteria tend to be multidrug resistant (MDR) ^10^. However, carbapenamase-producing enterobacteria are now common in low-income countries including Uganda ^11^.

Although pAmpC β-lactamase producing bacteria have been reported throughout the world ^3, 7, 12, 13^, there is little information about them in Uganda, especially their frequency among enterobacteria. Therefore, the aim of this study was to estimate the prevalence of pAmpC β-lactamase producing bacteria among Enterobacteriaceae isolated from individuals attending outpatient clinics in Kampala city and two rural districts in central Uganda. We show that pAmpC β-lactamase producing bacteria are prevalent in enterobacteriaceae isolated from urban and rural dwellers in Uganda, implying that ceftriaxone, an antibiotic commonly used to treat systemic infections in Uganda, could be associated with treatment failure.

## Methods

### Study setting

This cross-sectional study was conducted on flora from stool and urine of clients attending outpatients clinics in Kampala and two rural districts (Kayunga and Mpigi) in central Uganda ^14^. The study sites were purposively selected with an assumption that urban areas are associated with high bacterial carriage and exposure to antibiotics compared to rural areas ^15^. Kampala (urban) and Mpigi districts were assumed to have a wet-tropical climate, while Kayunga has a wet-dry tropical climate ^16^.

### Sample size estimation and sampling

The sample size for each study subsite was proportional to the contribution of the facility to the total outpatient clinic attendance in the months of April, May and June of 2006. All individuals attending the clinics were eligible to participate. By then, there was no data on antibiotic resistance among *E. coli* and/or *K. pneumoniae* isolates in well-defined community infections in Uganda. As such, the sample size was estimated based on an observed prevalence of 19.6% for *K. pneumoniae* carriage in clinical samples (urine, etc.) at Makerere University’s Clinical Microbiology Laboratory (unpublished observations). In each of the three districts, multistage sampling was done based on the average clinic attendance for the district. Thirty clusters of 16–20 participants were selected from each district using probability proportion to size sampling. Two busy days of a week were purposively chosen to visit a selected health care facility. When the number of participants exceeded 20, systematic sampling was done. A standardized interviewer-administered questionnaire was used to collect clinical and demographic data. Participants were instructed to provide stool or urine (if unable to provide stool) in a sterile screw-cap container. Samples from the rural districts were stored at 4°C for up to 24 hours prior to transportation, while those from Kampala were immediately transported to the laboratory at Makerere University College of Health Sciences for culturing.

### Culturing and identification of *E. coli* and *K. pneumoniae*

The procedure for culturing and isolate identification was described previously ^14^. Briefly, samples were streaked on MacConkey agar medium on the third/fourth quadrant, and incubated at 37°C for 18–24 hours in ambient air. In case of stool, samples were first emulsified in sterile normal saline before inoculation onto MacConkey agar plates. Lactose fermenting isolates with colony morphology suggestive of *E. coli* and *Klebsiella* spp. were subjected to oxidase testing and when negative, they were cultured for 18–24 hours on triple sugar and iron (TSI) agar, Simmons citrate agar, urea and Sulphide Indole Motility (SIM) medium for identification. Inconclusive isolates were confirmed as *E. coli* or *Klebsiella* by using the API 20E system (BioMerieux Marcy 1’Etoile, France).

### Antibiotic susceptibility testing

Antibiotic susceptibility testing (AST) was performed with the disc diffusion tests (DDT) on Mueller Hinton Agar (MHA) (Biolab, Hungary) as recommended by the Clinical Laboratory Standards Institute (CLSI) ^17^. Bacterial suspensions equivalent to 0.5 McFarland standard were prepared. The DDT included antibiotic disks (Biolab, Hungary) of ampicillin (10 µg), amoxicillin/clavulanate (20/10 µg), cefuroxime (30 µg), ceftriaxone (30 µg), cefotaxime (30 µg), ceftazidime (30 µg), meropenem (30 µg), sulfamethoxazole/trimethoprim (co-trimoxazole) (23.75/1.25 µg), chloramphenicol (30 µg), gentamicin (10 µg), ciprofloxacin (5 µg), nitrofurantoin (300 µg), cefepime (30 µg), piperacillin/tazobactam(100/10 µg) and cefoxitin (30 µg). *E. coli* ATCC25992, *Staphylococcus aureus*ATCC29213, *Pseudomonas aeruginosa* ATCC27853 and *Enterococcus faecalis* ATCC29212 were used as quality controls.

### Testing for AmpC β-lactamase production

Typically, AmpC β-lactamases confer resistance to cephamycins (e.g. cefoxitin), a characteristic widely used to distinguish them from the extended-spectrum β-lactamases (ESBLs), and to functionally screen for AmpC β-lactamase producing isolates ^3, 4, 18– 21^. As such, all cefoxitin resistant isolates in this study were screened by the cefoxitin/cloxacillin double-disc synergy test (CC-DDST) to detect AmpC β-lactamase production as previously described ^22^. As AmpC β-lactamases are associated with clavulanate resistance ^17, 23^, isolates with reduced susceptibility to amoxicillin/clavulanate were also tested for AmpC β-lactamase production. Susceptibility to cefoxitin and to third-generation cephalosporins was determined based on the CLSI guidelines (2007) ^24^. Furthermore, isolates that were positive on the CC-DDST (i.e. AmpC β-lactamase producers) were re-tested with E-test strips containing cefotetan and cefotetan/cloxacillin (CN/CNI, AB BIODISC, Solna, Sweden). E-test screening was considered positive for AmpC β-lactamase production when MIC ratio for cefotetan / cefotetan/cloxacillin was ≥8 ^22^. Testing for ESBL production was carried out as described previously ^25^, on isolates with reduced zone diameters to third-generation cephalosporins. Briefly, isolates with zone diameters of 22 mm, 25 mm, 27 mm and 27 mm for discs of ceftazidime, ceftriaxone, cefotaxime and aztreonam respectively, were considered suspect for ESBL-production, which was subsequently confirmed by the double disc synergy test (DDST). Detection of ESBLs was The DDST for detection of ESBLs was performed by using amoxicillin/clavulanate disc in the center, and discs of ceftazidime, ceftriaxone, aztreonam, and cefotaxime placed 15–20 mm center-to-center from the amoxicillin-clavulanate disc. Extension of the zone of inhibition towards the clavulanate disc was indicative of ESBL-production.

### Screening for ESBL and pAmpC β-lactamase genes

All isolates testing positive for AmpC β-lactamase production on the CC-DDST were tested by polymerase chain reaction (PCR) for pAmpC gene carriage. Further, as pAmpC β-lactamase genes have also been detected in isolates with reduced susceptibility to third-generation cephalosporins, we tested isolates with inhibition zone diameters of ≤27 mm, ≤25 mm and ≤22 mm for cefotaxime, ceftriaxone and ceftazidime, respectively, for pAmpC gene carriage ^17, 23^. In-house multiplex PCRs targeting AmpC β-lactamase genes *bla*_CIT_ *, bla*_DHA_ *, bla*_MOX_ *, bla*_FOX_ *, bla*_EBC_, *bla*_ACC_ and *bla*_CMY-2_ were performed using published primers, Thermo-Fisher Taq DNA polymerase and PCR master-mixes, and conditions ^6, 26^. Amplification was performed in a 3Prime Mid-size thermocycler (Techne, UK) and the expected amplicon sizes were successfully generated. PCR amplification of ESBL genes *bla*_CTX-M_, *bla*_TEM_ and *bla*_SHV_ was performed with Taq DNA polymerase (Thermo-Fisher Inc.) using published primers ^27^. Amplicons were sequenced (ACGT, Wheeling, IL, USA) by the chain termination method (Sanger sequencing) and sequences confirmed through BLAST-searching at NCBI. Phylogenetic group typing of *E. coli* was done according to the method of Clermont et al, in which PCR of a combination of two genes ( *chuA* & *yjaA*) and an anonymous DNA fragment are used to classify strains ^28^.

### Data analysis

The data were double entered for validation using EPIDATA software version 3.1, cleaned and exported to STATA (v14) for analysis. Data were compared across the districts using descriptive statistics, frequencies and bivariate analyses (cross-tabulations). Associations between outcome variables, i.e. isolates with ESBL/pAmpC genes, and categorical independent variables, i.e. socio-demographics, use of antibiotics, history of hospital admission and medical procedures three months prior to visits, were tested using Pearson’s Chi-square. A significant level was set at p<0.05. Similarly, odds ratios (ORs) between the categorical independent variables and outcome variables were determined. Variables with p<0.2 at bivariate analysis were entered into multivariate logistic regression models with backward elimination. Independent variables used were gender (male vs. female), health center level, health sub-district and district, history of admission, history of medical procedures and antibiotic use recalled by client and from health record during the previous three months. To control for the effect of clustering, regression with robust standard errors was used.

### Ethical statement

The study protocol and consent procedure were reviewed and approved by the Research Ethics Committee and the Higher Degrees committee of Makerere University Medical School (IRB #-2006-009) and the Uganda National Council for Science and Technology (HS246). All adult participants and guardians gave written informed consent before participation. The consent process included storage and use of the collected stool and urine samples for further studies. We obtained assent from participants below the age of 18 years in addition to informed consent from their parent/guardians/caregivers.

## Results

### Demographic characteristics, bacterial isolates

Of the 1,448 participants we enrolled, females were the majority i.e. 63.3% (913/1,448). Thirty three percent of the participants (474/1,448) were from Kampala, 35% (508/1,448) from Kayunga and 32% (466/1,448) from Mpigi. Around 56% (802/1,448) of the participants were in the 15-44-year age group (Table S1, *Extended data* ^29^).

From the 730 stool and 718 urine samples processed, 985 enterobacteria were isolated, of which 94.4% (930/985) were *E. coli* and 5.6% (55/985) were *K. pneumoniae*. Per district, 58% (274/474) of the enterobacteria were from Kampala, 80% (406/508) from Kayunga, and 65.5% (305/466) from Mpigi. The characteristics of the participants whose samples grew *E. coli* and *K. pneumoniae* are shown in Table S2 (see *Extended data* ^29^). None of the urine samples grew bacteria at ≥10 ^4 ^colony forming units (CFU) per milliliter implying that there was no infection-related growth. Overall, 37% (535/1,448) of the participants visited outpatient clinics for general conditions and bacteria grew in 68% (363/535) of these participants. Of the 731 participants who presented with infectious conditions, 67% (488/731) had bacterial growth in their samples. Of the 122 participants who visited HIV/AIDS clinics for routine checks, 72% (88/122) had growth (Tables S1 and S2, *Extended data* ^29^). Furthermore, 1,093 participants reported to have taken antibiotics three months prior to the visit, of whom 69% (755/1,093) had growth. Of the 125 participants who reported to have been previously admitted to hospitals, 62% (78/125) had growth. Of the 130 participants who reported to have undergone medical procedures three months prior to the clinic visit, 55% (71/130) had growth (Tables S1 and S2, *Extended data* ^29^).

### Prevalence of AmpC β-lactamase producing isolates

**Figure 1.**
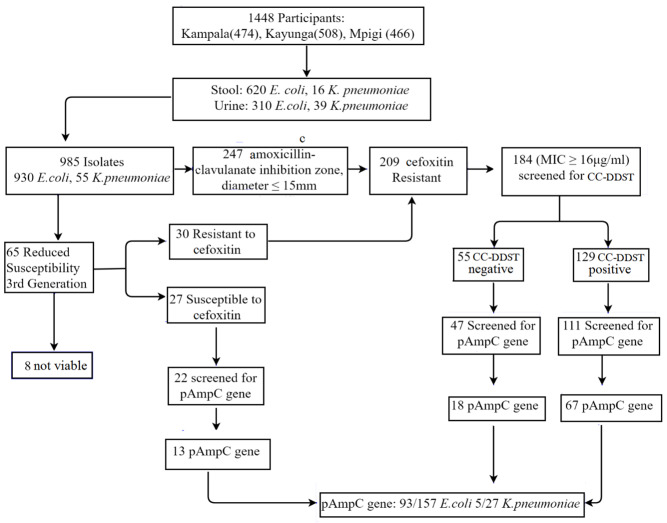
Study flow chart.

Of the 985 bacterial isolates investigated, 21% (209/985) were cefoxitin resistant. However, 25 cefoxitin resistant isolates were not available at the time of analysis, leaving 184 isolates that were investigated, of which 70% (129/184) were AmpC β-lactamase producers, while 30% (55/184) were non-producers ( Figure 1). Therefore, the prevalence of AmpC β-lactamase producers among cefoxitin resistant isolates was 70% (129/184), implying the overall prevalence of AmpC β-lactamase producing isolates among enterobacteria was 13.1% (129/985); 12.5% (116/930 *E. coli* and 23.6% (13/55) *Klebsiella*. Per district the prevalence of AmpC β-lactamase producing bacteria was 23.7% (65/274) Kampala, 12.1% (37/305) Mpigi, and 6.7% (27/406) Kayunga. Furthermore, given the association between AmpC β-lactamases and clavulanate resistance, 247 amoxicillin/clavulanate resistant isolates in this study (see below) comprising of 229 *E. coli* and 18 *Klebsiella,* were tested for cefoxitin resistance, majority of which i.e. 84.6% (209/247) were found to be cefoxitin resistant while only 15.4% (38/247) were cefoxitin susceptible. Of the 209 amoxicillin/clavulanate and cefoxitin resistant isolates, 61.7% (129/209) were AmpC producers ( Figure 1).

### Prevalence of pAmpC β-lactamase genes

Of 129 cefoxitin resistant and AmpC β-lactamase producing isolates, 111 were tested for pAmpC β-lactamase gene carriage. Of these, 60% (67/111) carried pAmpC genes. Furthermore, 47 of the 55 AmpC β-lactamase non-producers (see above) were tested for pAmpC gene carriage as they were cefoxitin resistant (MIC ≥16 μg/ml). Of these, 38% (18/47) carried pAmpC genes. Therefore, 54% (85/158) of the cefoxitin resistant isolates in this study (111 AmpC β-lactamase producers plus 47 AmpC β-lactamase non-producers) carried pAmpC β-lactamase genes. Isolates with reduced susceptibility to third-generation cephalosporins are suspects for AmpC β-lactamase production; in this study, 33.8% (22/65) of such isolates were cefoxitin susceptible, of which 59% (13/22) carried pAmpC genes. Overall, a total of 180 isolates (158 cefoxitin resistant plus 22 cefoxitin susceptible with reduced susceptibility to third-generation cephalosporins), comprising of 157 *E. coli* and 23 *Klebsiella*, were tested for pAmpC gene carriage ( Figure 1). Of these, 54% (cefoxitin resistant isolates, 85/158) were pAmpC positive while 59.1% (cefoxitin susceptible isolates with reduced susceptibility to third-generation cephalosporins, 13/22) were pAmpC positive, giving a total of 98 pAmpC positive isolates detected.

The overall prevalence of pAmpC genes in enterobacteria was 10% (98/985); by district it was 16.4% (45/274) in Kampala, 6.2% (25/406) Kayunga and 9.2% (28/305) Mpigi; hence, the urban district of Kampala had more pAmpC gene positive bacteria. Per species the prevalence of pAmpC genes among cefoxitin resistant and/or amoxicillin/clavulanate resistant isolates was 59% (93/157) in *E. coli* and 26.1% (5/23) *in Klebsiella*. pAmpC β-lactamase gene carriage correlated with AmpC β-lactamase production (χ ^2^ =11.7, P-value 0.0003). The pAmpC β-lactamase producing *E. coli* belonged to phylogenetic groups A (n=23), B1 (n=10), B2 (n=35) and D (n=25). Overall, 39.6% (44/111) of AmpC β-lactamase producing isolates did not carry pAmpC genes, of which eight were *Klebsiella* that do not carry chromosomal AmpC genes. The AmpC β-lactamase producing isolates of *E. coli* that were pAmpC negative were assumed to be hyper-producers of chromosomal AmpC β-lactamases. Relatedly, the AmpC β-lactamase producing isolates of *Klebsiella* that were pAmpC negative likely carried genes we did not screen for. The characteristics of participants who carried pAmpC gene-positive bacteria are shown in Table 1.

**Table 1.** Characteristics of participants who carried pAmpC gene positive bacteria.

Characteristics	pAmpC		
	Not present, n (%)	Present, n (%)	p-value
**Age group**			
0–14	260 (31.8)	36 (36.7)	0.131
15–44	440 (53.9)	55 (56.1)	
45+	117 (14.3)	7 (7.2)	
**Sex**			
Female	544 (66.5)	59 (60.2)	0.214
Male	274 (33.5)	39 (39.8)	
**Health center level**			
National referral	75 (9.1)	18 (18.4)	0.023
General hospital	220 (26.8)	28 (28.6)	
Health center IV	134 (16.3)	11 (11.2)	
Health center III	392 (47.8)	41 (41.8)	
**District**			
Kampala	198 (24.1)	44 (44.9)	<0.001
Kayunga	363 (44.2)	25 (25.5)	
Mpigi	260 (31.7)	29 (29.6)	
**Reason for visit**			
ISS	76 (9.8)	5 (5.3)	0.350
Infection	398 (51.2)	50 (52.6)	
General	303 (39.0)	40 (42.1)	
**History of admission**			
No	748 (92.4)	86 (88.7)	0.207
Yes	62 (7.6)	11 (11.3)	
**History of medical** **procedures**			
Contact	4 (7.3)	1 (14.2)	(omitted)
Inoculation	40 (72.7)	3 (42.9	
Surgery	11 (20.0)	3 (42.9	
**Use of antibiotics**			
No	194 (23.6)	24 (24.5)	(omitted)
Yes	627 (76.4)	74 (75.5)	
**Use of penicillin**			
No	427 (67.8)	53 (73.6)	0.313
Yes	203 (32.2)	19 (26.4)	
**Use of ciprofloxacin**			
No	566 (89.8)	59 (80.8)	0.020
Yes	64 (10.2)	14 (19.2)	
**Use of cotrimoxazol** **(septrin)**			
No	239 (34.3)	34 (40.0)	0.297
Yes	458 (65.7)	51 (60.0)	

ISS, immune suppression syndrome (HIV/AIDS).

**Figure 2.**
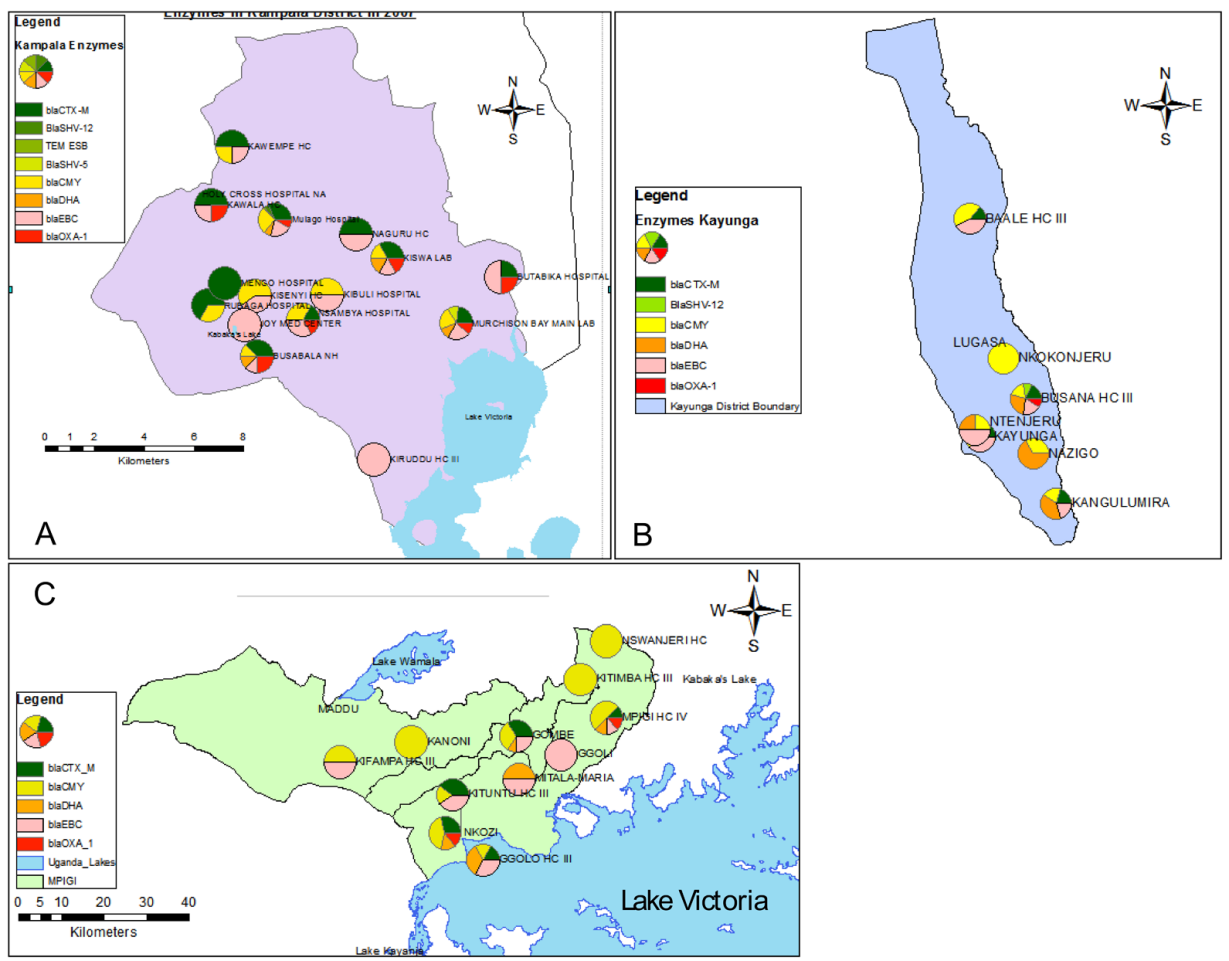
Distribution of β-lactamase genotypes in the three districts of Kampala, Kayunga and Mpigi.

The pAmpC genes detected were *bla*_CIT_ (n=54), *bla*_CMY-2_ (n=23), *bla*_CMY-4_ (n=31), *bla*_EBC_ (n=51, mainly *bla*_ACT-1_) and *bla*_DHA_ (n=20), Table 2. Twenty-two isolates carried ≥2 pAmpC genes and the most frequent combination was *bla*_EBC_ plus *bla*_CIT_. Furthermore, in this study, 11 ESBL producing isolates carried *bla*_CTX-M-15 _while three carried *bla*_CTX-M-28_. Co-existence of pAmpC with betalactamase genes ( *bla*_CTX-M_, *bla*_SHV_, *bla*_TEM_) occurred in 49% (48/98), 9.2% (9/98) and 34.7% (34/98) isolates, respectively. Figure 2 depicts the distribution of β-lactamase genotypes in the three districts.

**Table 2.** Prevalence of pAmpC β-lactamase genes among *E. coli* and *Klebsiella* across the three districts.

pAmpC gene ^a^	Specimen type (N=98)		District			Total ^b^
	Stool 55 (56%)	Urine 43 (44%)	Kampala 45/274 (16.4%)	Kayunga 25/406 (6.2%)	Mpigi 28/305 (9.2%)	
*bla*_CIT_	30	24	25 (13/12)	12 (2/10)	17 (8/9)	54
*bla*_DHA_	10	10	5	8	7	20
*bla*_EBC_	28	23	28	11	12	51

^a^Based on a multiplex PCR by Perez-Perez and Hanson, 2002; ^b^Numbers are derived from specimen type.

### Antibiotic resistance patterns

Generally, cefoxitin resistance varied across the three districts and the variation was statistically significant (p=0.023), Table S3 (see *Extended data* ^29^). Furthermore, 59% (58/98) of the pAmpC β-lactamase producing isolates were resistant to a β-lactam antibiotic and to two other classes of commonly used non-β-lactam antibiotics, implying they were MDR ^30^. Two of the isolates were resistant to co-trimoxazole, ciprofloxacin, gentamicin, nitrofurantoin and chloramphenicol, while seven were co-resistant to these drugs excluding nitrofurantoin, and one was resistant to the same drugs except chloramphenicol. Resistance to ciprofloxacin, co-trimoxazole and gentamicin was noted in nine isolates, co-trimoxazole and chloramphenicol in 22 and co-trimoxazole and gentamicin in five. Resistance to individual antibiotics among the pAmpC β-lactamase gene positive isolates was as follows, co-trimoxazole 79% (77/98); chloramphenicol 34.5% (34/98); ciprofloxacin 28% (27/98); gentamicin 23.5% (23/98); nitrofurantoin 8% (8/98); piperacillin/tazobactam 30.6% (30/98). None of the isolates were resistant to carbapenems.

### Factors associated with carriage of pAmpC β-lactamase producing bacteria

There was significant association between health center level, district of residence, use of ciprofloxacin and sample type with carriage of pAmpC β-lactamase producing bacteria, Table 3. After adjusting for each of them, the district of residence remained an independent risk factor for carriage of pAmpC β-lactamase producing bacteria, Table 3. Overall, we found that residing in a rural district (Kayunga/Mpigi) was associated with low carriage of pAmpC β-lactamase producing bacteria (aOR 0.23 (95% CI:0.11, 0.47) and aOR 0.49 (95% CI:0.25, 0.99), respectively). Similarly, participants who were 45 years and above carried less pAmpC positive bacteria (aOR 0.17; 95% CI:0.05, 0.62). Ciprofloxacin use was an independent risk factor for carriage of pAmpC positive bacteria (aOR 2.61; 95% CI:1.28, 5.32), Table 3.

**Table 3.** Factors associated with carriage of pAmpC β-lactamase producing bacteria.

Characteristic	pAmpC gene				
	Not present, n (%)	Present, n (%)	p-value	cOR (95% CI)	aOR (95% CI)
**Age group**					
0–14	260 (31.8)	36 (36.7)	0.131	1.0	1.0
15–44	440 (53.9)	55 (56.1)		0.90 (0.58, 1.41)	0.59 (0.32, 1.07)
45+	117 (14.3)	7 (7.2)		0.43 (0.19, 1.00) *	0.17 (0.05, 0.62) *
**Gender**					
Female	544 (66.5)	59 (60.2)	0.214	1.0	1.0
Male	274 (33.5)	39 (39.8)		1.31 (0.85, 2.02)	1.48 (085, 2.56)
**Health center level**					
National Referral	75 (9.1)	18 (18.4)	0.023	1.0	1.0
General Hospital	220 (26.8)	28 (28.6)		0.53 (0.28, 1.01)	1.04 (0.40, 2.70)
Health Center IV	134 (16.3)	11 (11.2)		0.34 (0.15, 0.76) *	1.53 (0.47, 5.00)
Health Center III	392 (47.8)	41 (41.8)		0.44 (0.24, 0.80) *	0.74 (0.28, 1.98)
**District**					
Kampala ^a^	198 (24.1)	44 (44.9)	<0.001	1.0	1.0
Kayunga ^b^	363 (44.2)	25 (25.5)		0.31 (0.18, 0.52)	0.23 (0.11, 0.47) *
Mpigi ^c^	260 (31.7)	29 (29.6)		0.50 (0.30, 0.83) *	0.49 (0.25, 0.99) *
**Reason for visit**					
ISS	76 (9.8)	5 (5.3)	0.350	1.0	1.0
Infection	398 (51.2)	50 (52.6)		1.91 (0.74, 4.94)	2.60 (0.73, 9.32)
General	303 (39.0)	40 (42.1)		2.01 (0.77, 5.26)	3.20 (0.86, 11.86)
**History of admission**					
No	748 (92.4)	86 (88.7)	0.207	1.0	1.0
Yes	62 (7.6)	11 (11.3)		1.54 (0.78, 3.04)	0.55 (0.21, 1.49)
**History of medical procedures**					
Contact	4 (7.3)	1 (14.2)	0.178		
Inoculation	40 (72.7)	3 (42.9			
Surgery	11 (20.0)	3 (42.9			
**Antibiotic use (any)**					
No	194 (23.6)	24 (24.5)	0.850		
Yes	627 (76.4)	74 (75.5)			
Use of penicillin					
No	427 (67.8)	53 (73.6)	0.313		
Yes	203 (32.2)	19 (26.4)			
Use of ciprofloxacin					
No	566 (89.8)	59 (80.8)	0.020	1.0	1.0
Yes	64 (10.2)	14 (19.2)		2.10 (1.11, 3.97)	2.61 (1.28, 5.32)
Use of co-trimoxazole					
No	239 (34.3)	34 (40.0)	0.297		
Yes	458 (65.7)	51 (60.0)			

cOR, crude odds ratio; aOR, adjusted odds ratio; ISS, immune suppression syndrome (HIV/AIDS); ^*^Statistically significant association.

## Discussion

AmpC β-lactamases are clinically important in that community-acquired infections arising from strains producing these enzymes may not respond to empiric treatment with common antibiotics. The cefoxitin/cloxacillin double-disc synergy screening of cefoxitin and amoxicillin/clavulanate resistant isolates for AmpC β-lactamase production is a simple and efficient method of quickly detecting these resistance mechanisms in isolates ^21, 22^. Using this approach, the prevalence of AmpC β-lactamase producing bacteria in this study (13.2%), and the prevalence of pAmpC β-lactamase gene carriage (26-59%) was high but comparable to the rate of 36.5% at Mbarara Regional Referral Hospital in South-western Uganda ^20^. The prevalence in our study is higher than rates from other East African settings ^31– 33^; however, the study populations were varied, making direct comparison difficult. Furthermore, in this study, co-carriage rates of ESBL- and pAmpC genes was high, which is a cause for concern as individuals carrying strains producing these enzymes could be reservoirs of spread for MDR bacteria ^34, 35^. A significant number of isolates that were susceptible to third-generation cephalosporins but cefoxitin resistant carried pAmpC genes, a discordance that has been reported before ^3, 36^. The overall carriage rate (10%) for pAmpC genes in enterobacteriaceae in this study reflects extensive use of antibiotics, as found in Libya where a fecal carriage rate of 6.7% for pAmpC β-lactamase producing bacteria in the community was reported ^37^. Importantly, ceftriaxone has been used in Uganda for the last two decades, mainly in empirical treatment of systemic bacterial infections and currently its prescription among in-patients is higher than that of other antibiotics ^38^. Such extensive use of ceftriaxone could be a driving force behind the high pAmpC gene carriage rates. Coexistence between *bla*_EBC_ and *bla*_CIT_ genes in isolates has been reported before in Africa and Asia ^36, 39^. *bla*_CIT_ reported in this study comprised of the *bla*_CMY-2_ and *bla*_CMY-4_ genes. In Africa, *bla*_CMY-4_ was first reported in North Africa. The detection of *bla*_DHA_ and *bla*_EBC_ genes is of concern as *bla*_ACT-1_, a prototype gene for the *bla*_EBC_ and *bla*_DHA_ genes, is linked to a functional *ampR* regulator and is inducible ^40, 41^. In Seoul, Korea, four of the five deaths from bloodstream infections due to *bla*_DHA_ producing *K. pneumoniae* were associated with treatment with extended spectrum cephalosporins ^42^.

In this study, about half of the isolates exhibiting reduced susceptibility to third-generation cephalosporins carried pAmpC β-lactamase genes, which contrasted findings from Northern Europe where 100% of isolates exhibiting reduced susceptibility to third-generation cephalosporins carried pAmpC genes ^23^. Furthermore, 73% of cefoxitin resistant and pAmpC positive isolates were susceptible to third-generation cephalosporins, in contrast with 26% (10/38) reported from Switzerland, for pAmpC producing isolates that were susceptible to third-generation cephalosporins ^43^. Overall, findings in this study suggest that antibiotic susceptibility testing of enterobacteria in Uganda may yield false results for third-generation cephalosporins e.g. ceftriaxone, cefotaxime and ceftazidime. Given that bacterial isolates are not routinely tested for AmpC β-lactamase production, region specific protocols guided by surveillance data are necessary.

In *E. coli*, phylogenetic group analysis has been used to differentiate virulent/extra-intestinal strains, which predominantly belong to phylogenetic groups B2 and D, from commensal strains that belong to groups A and B1 ^28^. In this study, the predominance of groups B2 and D (n=60) compared to groups A and B1 (n=33) in the community is cause for concern as they are associated with pathogenicity, implying that strains with potential to cause extra-intestinal disease are prevalent, supporting the notion that occurrence of pAmpC β-lactamase producing strains in the community is of public health concern ^44, 45^. One limitation in this study was that we were not able to genotype isolates with internationally acceptable procedures like the multilocus sequence typing (MLST) to determine the sequence types.

## Conclusions

AmpC β-lactamase production and pAmpC β-lactamase encoding genes are prevalent among *E. coli* and *K. pneumoniae* isolates from urban and rural dwellers in Uganda. As pAmpC genes are easily transferrable between species and have been associated with outbreaks of community- and hospital-acquired infections, pAmpC beta-lactamase producing bacteria may represent a threat in low-income settings. There is need for testing for cefoxitin resistance during routine antibiotic susceptibility testing, especially among isolates that are resistant to amoxicillin/clavulanate, as well as isolates that are susceptible to third-generation cephalosporins.

## Data availability

### Underlying data

Figshare: Underlying data for manuscript “Prevalence of plasmid-mediated AmpC beta-lactamases in Enterobacteria isolated from urban and rural folks in Uganda” by Najjuka FC *et. al*. https://doi.org/10.6084/m9.figshare.13259984 ^29^.

This project contains the following underlying data:

- Participants_recruited_from_outpatient_clinics.xls (participant data underlying Table S1)
- Participants_whose_samples_had_growth.xls (participant data underlying Table S2)
- Susceptibility profile of PampC-isolates-09092020-1.xls (antibiotic susceptibility data underlying Table S3)
- Table of cefoxitin resistant-susceptible Isolates.xls (cefoxitin resistance data underlying Table S3)

### Extended data

Figshare: Extended data for manuscript "Prevalence of plasmid-mediated AmpC beta-lactamases in Enterobacteria isolated from urban and rural folks in Uganda" by Najjuka FC *et. al*. https://doi.org/10.6084/m9.figshare.13259984 ^29^.

This project contains the following extended data:

- Table S1.docx (Characteristics of participants attending OPDs in the three study sites)
- Table S2.docx (Characteristics of participants with bacterial growth on culturing (n=985))
- Table S3.docx (Antibiotic resistance rates among E. coli and Klebsiella isolated from individuals attending outpatient clinics in Kampala, Kayunga and Mpigi districts (2007–2008))

Data are available under the terms of the Creative Commons Zero "No rights reserved" data waiver (CC0 1.0 Public domain dedication).
