# Supplementary material for: Prevalence of plasmid-mediated AmpC beta-lactamases in Enterobacteria isolated from urban and rural folks in Uganda
Source: AAS Open Res. 2020 Nov 30;3:62. [Version 1] doi: 10.12688/aasopenres.13165.1 (PMC8422338; doi:10.12688/aasopenres.13165.1)
Supplement: Peer review history

# Peer review: Reviewer response for version 1

**DOI:** 10.21956/aasopenres.14270.r28822

**Published:** 2021-09-06

**Authors:** Muhammad Usman Qamar

**Affiliations:** 1 Department of Microbiology, Faculty of Life Sciences, Government College University, Faisalabad, Pakistan

The authors did a comprehensive study on the prevalence of AMR in animals. This topic is the need of the hour. Overall, the study is appropriate and should be accepted for indexing. 

1. Change the word Enterobacteriaceae to Enterobacterales. 
2. Review the English grammar mistakes
3. The Discussion part should be more elaborate. 

Is the work clearly and accurately presented and does it cite the current literature?

Yes

If applicable, is the statistical analysis and its interpretation appropriate?

Yes

Are all the source data underlying the results available to ensure full reproducibility?

Yes

Is the study design appropriate and is the work technically sound?

Yes

Are the conclusions drawn adequately supported by the results?

Yes

Are sufficient details of methods and analysis provided to allow replication by others?

Yes

Reviewer Expertise:

I have been working on One Health AMR agenda. I am mainly working on molecular epidemiology of AMR, characterization, and risk factors associated with AMR.

I confirm that I have read this submission and believe that I have an appropriate level of expertise to confirm that it is of an acceptable scientific standard, however I have significant reservations, as outlined above.

# Peer review: Reviewer response for version 1

**DOI:** 10.21956/aasopenres.14270.r28846

**Published:** 2021-09-06

**Authors:** Samuel Kariuki, Robert Onsare

**Affiliations:** 1 Centre for Microbiology Research, Kenya Medical Research Institute, Nairobi, Kenya; 2 Kenya Medical Research Institute (KEMRI), Nairobi, Kenya

The study was aimed at estimating the prevalence of pAmpC β-lactamase producing bacteria among Enterobacteriaceae isolated from individuals attending outpatient clinics in Kampala city and two rural districts in central Uganda.

 The study found that pAmpC beta-lactamase producing enterobacteria are prevalent in urban and rural dwellers in Uganda and recommended that cefoxitn should be considered during routine susceptibility testing in this setting.

 The article is well written, with appropriate citations and the methodology adequately answers the study questions. The conclusions made are adequately supported by the results.

Is the work clearly and accurately presented and does it cite the current literature?

Yes

If applicable, is the statistical analysis and its interpretation appropriate?

Yes

Are all the source data underlying the results available to ensure full reproducibility?

Yes

Is the study design appropriate and is the work technically sound?

Yes

Are the conclusions drawn adequately supported by the results?

Yes

Are sufficient details of methods and analysis provided to allow replication by others?

Yes

Reviewer Expertise:

Epidemiology of infectious diseases and antimicrobial resistance of tropical infections

We confirm that we have read this submission and believe that we have an appropriate level of expertise to confirm that it is of an acceptable scientific standard.

# Peer review: Reviewer response for version 1

**DOI:** 10.21956/aasopenres.14270.r28723

**Published:** 2021-07-07

**Authors:** Soo Tein Ngoi

**Affiliations:** 1 Department of Medical Microbiology, University Malaya, Kuala Lumpur, Malaysia

The study presented a detailed analysis of the prevalence of AmpC beta-lactamase producers, pAmpC and ESBL genes in the two most common enteric bacterial species, *E. coli *and *K. pneumoniae*. The authors observed a relatively higher prevalence of AmpC producers in urban areas than in rural areas. Detailed molecular characterization was performed to identify the pAmpC and ESBL genotypes, and a great diversity of the beta-lactamase genes was observed among the strains. Moreover, the common carriage of beta-lactamase-producing and potentially virulent *E. coli *strains in the population indeed poses a great threat to public health, not only causing infections in susceptible hosts but also serves as a reservoir for spreading antimicrobial resistance genes among the pathogenic bacteria. This study adds knowledge to the molecular epidemiology of beta-lactamase-producing enteric bacteria, which is one of the main concerns pertaining to the rising trend of antimicrobial-resistant organisms. However, the authors should make the following changes:

1.  The sampling design and procedures, bacterial isolation, antimicrobial susceptibility testing, and phenotypic detection of AmpC and ESBL producers have already been reported in detail in the previous article published by the authors ^1^. Please remove the related sections in Methods and Results to avoid duplication of data. I suggest the authors include a summary of the previous findings in the last paragraph of the Introduction since this study is considered an extension of the previously reported work.  
2. Figure 2 clearly depicts the distribution of the different *bla *genotypes according to geographical regions. This is very interesting as there seems to be a predominance of specific *bla *genes in the different districts. Could the authors elaborate more on this finding? Statistical analysis could be done to determine if the correlation between the genotypes and the geographical regions is significant or not. Also, the figure legends should be standardized to ease viewing.
3. Ciprofloxacin use was found to be a risk factor for the carriage of pAmpC-positive strains. It would be of interest if the authors could discuss the use of fluoroquinolones in the region.    

Minor comments:

1.  Please standardize the nomenclature of *K. pneumoniae* throughout the text.
2.  The family name, *Enterobacteriaceae*, should be capitalized and italicized.

Is the work clearly and accurately presented and does it cite the current literature?

Partly

If applicable, is the statistical analysis and its interpretation appropriate?

I cannot comment. A qualified statistician is required.

Are all the source data underlying the results available to ensure full reproducibility?

Yes

Is the study design appropriate and is the work technically sound?

Yes

Are the conclusions drawn adequately supported by the results?

Yes

Are sufficient details of methods and analysis provided to allow replication by others?

Yes

Reviewer Expertise:

Antimicrobial resistance; bacterial genomics; molecular epidemiology

I confirm that I have read this submission and believe that I have an appropriate level of expertise to confirm that it is of an acceptable scientific standard, however I have significant reservations, as outlined above.

## References

[1] : Antimicrobial susceptibility profiles of Escherichia coli and Klebsiella pneumoniae isolated from outpatients in urban and rural districts of Uganda.*BMC Res Notes*.2016;9: 10.1186/s13104-016-2049-823510.1186/s13104-016-2049-827113038PMC4843195

# Response

**Published:** 2021-08-16

**Authors:** David Kateete

**Affiliations:** Makerere University, Uganda

**Response to comments:**

 Thank you for your insightful comments. We are using these to improve our manuscript.

 Please find below, our response to each of the raised concerns. Kindly note that we are awaiting an additional reviewer's report to upload the revised manuscript hence, the changes to the manuscript in light of your suggestions will be seen when we upload the revised manuscript.

**Comment 1:**

 The sampling design and procedures, bacterial isolation, antimicrobial susceptibility testing, and phenotypic detection of AmpC and ESBL producers have already been reported in detail in the previous article published by the authors ^1^. Please remove the related sections in Methods and Results to avoid duplication of data. I suggest the authors include a summary of the previous findings in the last paragraph of the Introduction since this study is considered an extension of the previously reported work.

**Response:**

 We agree. We have revised the Introduction accordingly.

**Comment 2:**

 Figure 2 clearly depicts the distribution of the different *bla *genotypes according to geographical regions. This is very interesting as there seems to be a predominance of specific *bla *genes in the different districts. Could the authors elaborate more on this finding? Statistical analysis could be done to determine if the correlation between the genotypes and the geographical regions is significant or not. Also, the figure legends should be standardized to ease viewing.

**Response:**

 Again, thanks for your appreciation of our analysis of the *bla* genotypes according to geographical distribution. We repeated the statistical analysis as advised; however, the results showed no statistically significant correlation between the genotypes and the geographical regions i.e., none of the enzymes was significantly in greater proportion compared to others across the different districts (p-value = 0.208). The figure legends are now standardized and easily viewable.

**Comment 3:**

 Ciprofloxacin use was found to be a risk factor for the carriage of pAmpC-positive strains. It would be of interest if the authors could discuss the use of fluoroquinolones in the region.   

**Response:**

 As advised, in our revised manuscript we have discussed the use of fluoroquinolones in Uganda and generally the East African region.

**Minor comments:**

- Please standardize the nomenclature of *K. pneumoniae* throughout the text.
- The family name, *Enterobacteriaceae*, should be capitalized and italicized.

**Response:**

 We have standardized the nomenclature of *Klebsiella pneumoniae*, and italicized the name *Enterobacteriaceae*. We thank you.
